# Social Learning in Vespula Germanica Wasps: Do They Use Collective Foraging Strategies?

**DOI:** 10.1371/journal.pone.0152080

**Published:** 2016-03-18

**Authors:** Mariana Lozada, Paola D’ Adamo, Micaela Buteler, Marcelo N. Kuperman

**Affiliations:** 1 INIBIOMA-CONICET, Universidad Nacional del Comahue, Quintral 1250, 8400 Bariloche, Argentina; 2 Consejo Nacional de Investigaciones Científicas y Técnicas, Bariloche, Argentina; 3 Centro Atómico Bariloche (CNEA) and Instituto Balseiro, 8400 Bariloche, Argentina; University of Sussex, UNITED KINGDOM

## Abstract

*Vespula germanica* is a social wasp that has become established outside its native range in many regions of the world, becoming a major pest in the invaded areas. In the present work we analyze social communication processes used by *V. germanica* when exploiting un-depleted food sources. For this purpose, we investigated the arrival pattern of wasps at a protein bait and evaluated whether a forager recruited conspecifics in three different situations: foragers were able to return to the nest (full communication), foragers were removed on arrival (communication impeded), or only one forager was allowed to return to the nest (local enhancement restricted). Results demonstrated the existence of recruitment in *V. germanica*, given that very different patterns of wasp arrivals and a higher frequency of wasp visits to the resource were observed when communication flow between experienced and naive foragers was allowed. Our findings showed that recruitment takes place at a distance from the food source, in addition to local enhancement. When both local enhancement and distant recruitment were occurring simultaneously, the pattern of wasp arrival was exponential. When recruitment occurred only distant from the feeder, the arrival pattern was linear, but the number of wasps arriving was twice as many as when neither communication nor local enhancement was allowed. Moreover, when return to the nest was impeded, wasp arrival at the bait was regular and constant, indicating that naive wasps forage individually and are not spatially aggregated. In conclusion, this is the first study to demonstrate recruitment in *V. germanica* at a distance from the food source by modelling wasps’ arrival to a protein-based resource. In addition, the existence of correlations when communication was allowed and reflected in tandem arrivals indicates that we were not in the presence of random processes.

## 1 Introduction

*Vespula germanica* is a social wasp that has become established outside its native range in many regions of the world, becoming a major pest in the invaded areas [1–5]. This species typically has an annual cycle and colonies grow at a fast rate during a short period of time during the summer and early autumn. Thus, efficient foraging is crucial for colony development. *V. germanica* wasps present very flexible foraging behavior, as they prey on other insects, feed on honeydew from aphids and fruit, and scavenge on carrion, garbage and food associated with human outdoor activities. Sociality can be an important component in achieving such efficient foraging behavior, since social communication greatly improves resource exploitation [6].

Within social communication, recruitment is an important mechanism which is widely observed in many hymenopteran species. For example, recruitment behaviors such as trophalaxis and the waggle dance have been much studied in honeybees [7–11], as have pheromone cues in ants and stingless bees [12–15]. Recruitment leads conspecifics to a certain site, facilitating efficient exploitation of temporary resources [6]. As resource quality and quantity vary in space and time, recruitment has been shown to be an advantageous strategy that helps social animals to increase their foraging effectiveness [16–21]. In social wasps, recruitment has not been studied in depth and research on the use of signals to recruit nest-mates has yielded mixed results [19–24]. Some studies have shown that intranidal olfactory cues acquired during trophalaxis orient foraging wasps [25–27]. Another type of recruitment strategy used by stingless bees involves pilot flights in which they guide recruits to the newly found resources [28]. This behavior has been described also for Vespula vulgaris.

Recent literature has shown that nest-based recruitment seems to occur in *Vespula vulgaris*[29] and *V. pennsylvanica* Vespid wasps [30]. In *Vespula germanica* few studies on the recruitment processes have been conducted [25, 31, 32]. For example, Overmyer and Jeanne [25] demonstrated the occurrence of nest-based recruitment for carbohydrate resources but suggested that wasps are not able to recruit to protein sources. Interspecific differences in foraging mechanisms within the genus warrant further experiments to investigate recruitment in social wasps [29].

Previous studies on *V. germanica* foraging behavior have shown that this wasp displays numerous learning abilities while relocating protein-based resources (e.g. [33] for a review). These investigations, which revealed the existence of diverse cognitive mechanisms in forager wasps scavenging on stable un-depleted sources, have focused mainly on individual cognition involving the learning of visual, odor and spatial cues [34–38]. For instance, these studies demonstrated that one successful foraging experience seems to be sufficient to establish an association between diverse cues and food reward [39–41]. In the same line, after foraging on an un-depleted resource, foragers perform circling flights over it, to learn positional cues for their return (e.g. [42, 43]). The number of these flights is significantly reduced after just one feeding visit [44], as was also observed in honeybees [45]. Interestingly, different patterns of relocating behavior have been observed, depending on whether wasps were scavenging in habitats with scarce or abundant vegetation [39]. The above-mentioned studies did not consider the social component involved in each foraging situation, given that all wasps that approached the experimental array were removed, except for the marked individual under observation.

Cognitive abilities at a social level, which integrate individual capacities with collective strategies, have been little explored in *V. germanica* [17]. There is evidence that, in the nest, naive foragers of *V. germanica* and *V. vulgaris* learn the odor of a rich food and use that information to help locate the source of the odor in the field [25]. Vespula wasps are not known to mark food sources with odors [31, 46]. However, odor emitted by wasps is involved in foraging communication at the food source, leading to local enhancement [47] in *V. germanica* [16, 17] and other vespids [18, 27, 48]. D’Adamo and Lozada [16] demonstrated that local enhancement, a resource-based recruitment consisting in the attraction of foragers by conspecifics at a protein bait, was achieved through a combination of odor and visual cues.

The main goal of the present study was to gain further knowledge on recruitment processes used by *V. germanica*, and on how these may lead to an increase in exploitation efficiency of an un-depleted protein based resource. For this purpose, we investigated the arrival pattern of wasps to a protein bait and evaluated whether a forager can recruit conspecifics. We studied the corresponding time series to find patterns indicating correlations associated to recruitment in form of trains of arrivals and the departure from a constant arrival rate characteristic of a Poisson random process.

## 2 Material and Methods

The experiments were conducted in natural, outdoor environments, near San Carlos de Bariloche (41°S, 71°W), Argentina, during the V. germanica wasp’s most active period (February–April) in 2010 and 2011. All experiments were carried out in public places, on pebble lake shores, on sunny days, under non-windy conditions. Experiments consisted in observing the behavior of foraging wasps arriving at a feeder with 20 g of ground bovine meat on a white plastic dish for a duration of thirty minutes. When free-flying wasps arrived at the feeder, a control protocol and two different treatments were carried out.

**No Treatment** The control consisted of allowing all wasps to arrive at the bait and leave. The time of each arrival was recorded. This treatment allowed both local enhancement and recruitment processes away from the resource to occur.

**Treatment 1** consisted in marking the first wasp that landed on the bait with washable paint for further identification according to D’Adamo and Lozada (2009). This wasp was allowed to collect food and fly back to the nest, and typically returned a few minutes later. The arrival of subsequent wasps was recorded and these wasps were removed with an aspirator, in order to let only one wasp return to the nest. In this treatment, communication between the marked wasp and the colony was allowed although local enhancement was reduced by aspirating all other wasps.

**Treatment 2** consisted in removing wasps as soon as they landed on the bait, in order to avoid local enhancement effects as well communication with the colony, given that all wasps were removed with an aspirator and could not return to the nest.

Each treatment was replicated 15 times. Each experiment had a duration of 30 minutes since the arrival of the first wasp to the feeder, defined as t = 0.

## 3 Data analysis

We analyzed the time series corresponding to the arrival of wasps to the feeder, to study the lapses between consecutive wasp arrivals and the cumulated number of visiting wasp. The differences between the three situations observed in our study could shed some light about plausibility of the existence of some type of communication between wasps which have previously visited the bait and other newcomer foragers.

After recording the arrival times of wasp visiting the feeder for each treatment and the control procedure, we performed an analysis tending to detect differences and similarities between them, focused on the the temporal pattern of arrivals, and also the distribution of lapses between consecutive arrivals.

For the analysis of total number of wasps arriving at the feeder compared among all treatments we used the Kruskal Wallis test. The comparison of the number of arrives between two treatments was done by means of the Mann Withney U tests.

In each case we performed a regression analysis to test the linear or non linear character of the data. When the arrival pattern exhibited a linear behavior, we estimated the arrival rate by least squares approximation. Each time series consisted of a small number of events, so we have performed a statistical analysis of several series. In order to statistically characterize this behavior we computed the time difference between consecutive arrivals for several experiments and constructed histograms representing the distribution of the duration of the intervals between arrivals.

We have also simulated the arrival of wasps as if corresponding to a Poisson process with adapted rate to compare the numerical and experimental results and discriminate between a correlated arrival process and a stochastic one.

## 4 Results

### 4.1 Total number of wasps

**Fig 1:**
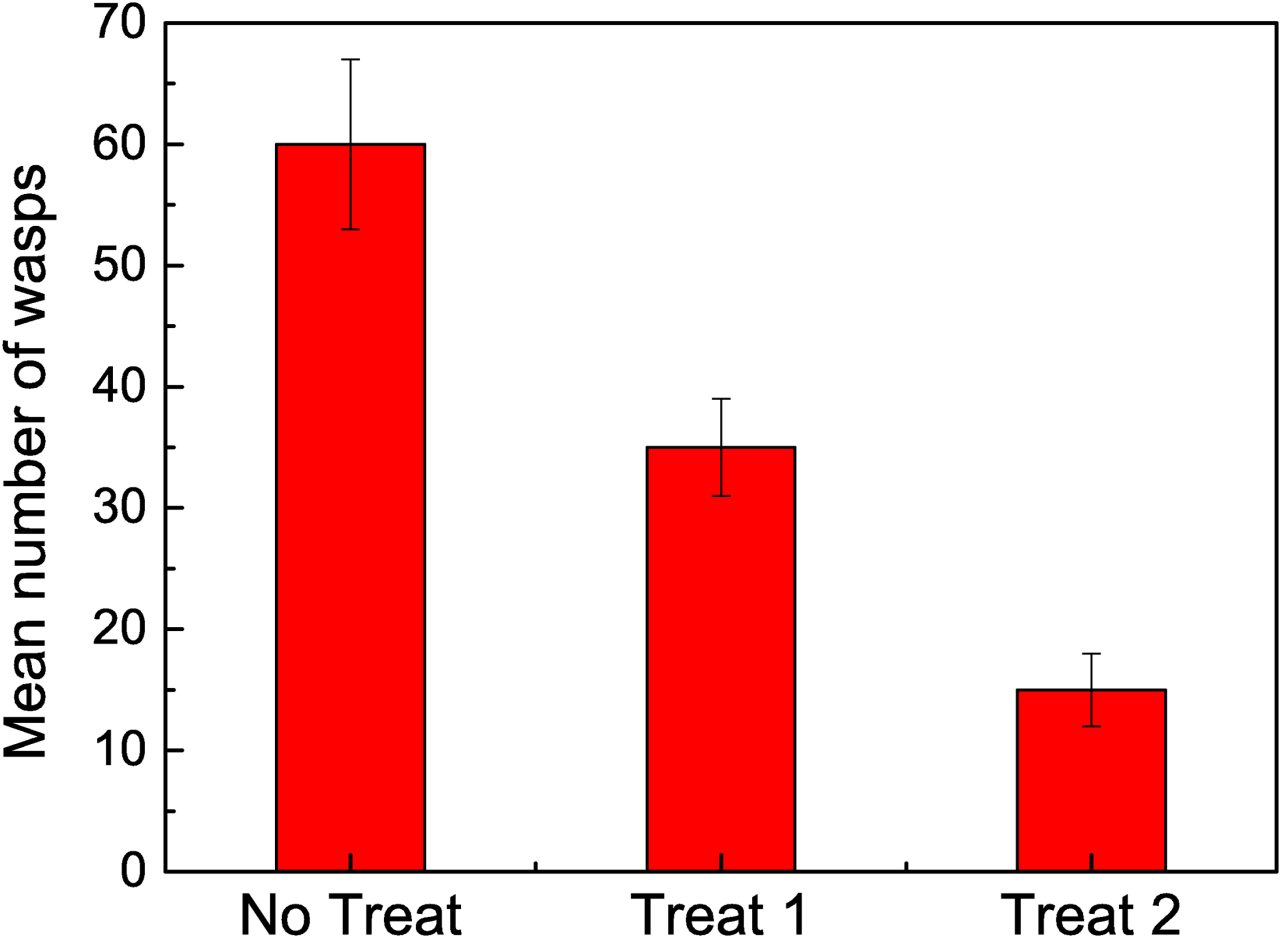
Mean number of arrived wasps within a 30 minute interval for each case: No Treatment, Treatment 1, Treatment 2.

When comparing the total number of wasps that arrived at the feeder, we found significant differences between the three groups (*χ*^2^ = 14.059, *df* = 2, *p* < 0.001). Moreover, the comparison between no treatment and treatment 1 also showed significant differences (*z* = 2.91, *p* < 0.01), as well as for the comparison between no treatment and treatment 2 (*z* = 3.98, *p* < 0.0001), and for the comparison between treatments 1 and 2 (*z* = 2.84, *p* < 0.05). When no recruitment was allowed, the number of arriving wasps was approximately 25% from the total number of wasps arriving when communication was allowed. The mean values are shown in Fig 1.

### 4.2 Time series analysis

#### No treatment (Communication allowed)

**Fig 2:**
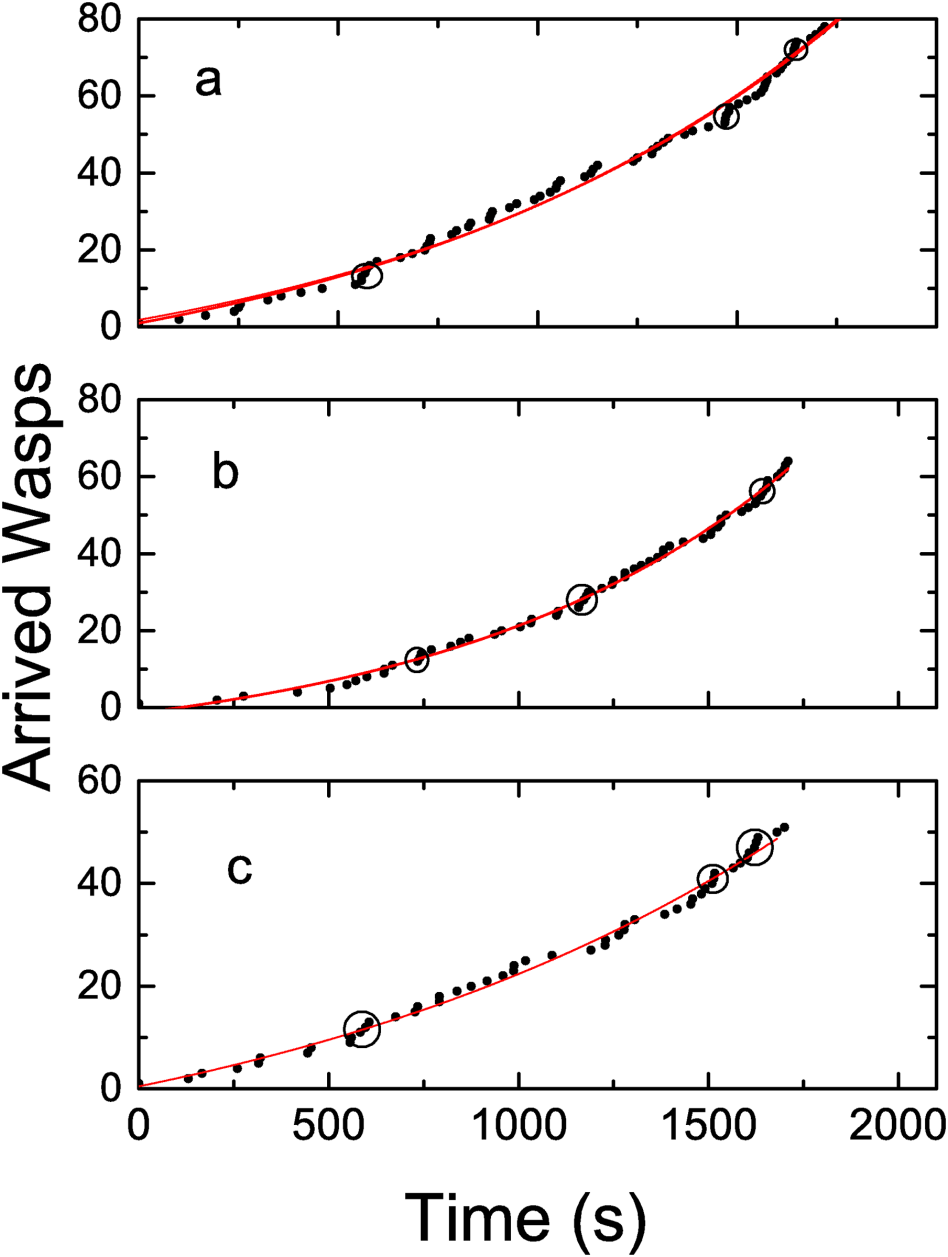
Cumulative number of arrived wasp in time. The full lines correspond to a fit using a growing exponential function (see text). The exponents, in unities of s^−1^ are (a) 8.68 10^−4^ ± 3.4 10^−5^, (b) 1.081 10^−3^ ± 4.3 10^−5^ (c)6.98 10^−4^ ± 6.4 10^−5^. Circles indicate trains of wasps arriving simultaneously. Each plot contains information about a different experiment, under similar conditions: sunny non-windy day.

When no treatment was applied, i.e. transfer of information was allowed, the time series of wasps arrival shows two interesting features: a non linear behaviour and the occurrence of trains of wasps that we defined as the arrival of three wasps in a lapse no longer than 8 seconds. Fig 2 illustrates three of these experiments, corresponding to the cases with the highest and lowest number of arrived wasps and an intermediate case. Each point in these plots corresponds to the arrival of a single wasp.All of them show a deviation from a linear behaviour. To test this, we performed regression analysis considering linear, quadratic and exponential fits. The deviation from a linear behaviour is apparent however, given the time duration of each experiment, we find no quantitative differences when fitting with a quadratic or exponential function. Though we have tested linear, quadratic and exponential fittings, the fitting displayed in Fig 2 correspond to the exponential one. The values of the adjusted coefficient of determination *R*^2^ calculated for each fit of the curves shown in Fig 2 are included in Table 1.

**Table 1:** Adjusted *R*^2^ values for a linear, quadratic and exponential fit of data shown in Fig 2.

Figure	Linear	Quadratic	Exponential
a	0.960	0.993	0.995
b	0.943	0.996	0.996
c	0.972	0.992	0.993

At the same time, we can verify the occurrence of trains of wasps arriving simultaneously to the feeder (arrival of three wasps in a lapse no longer than 8 seconds). Some of these trains are highlighted with circles in Fig 2.

**Fig 3:**
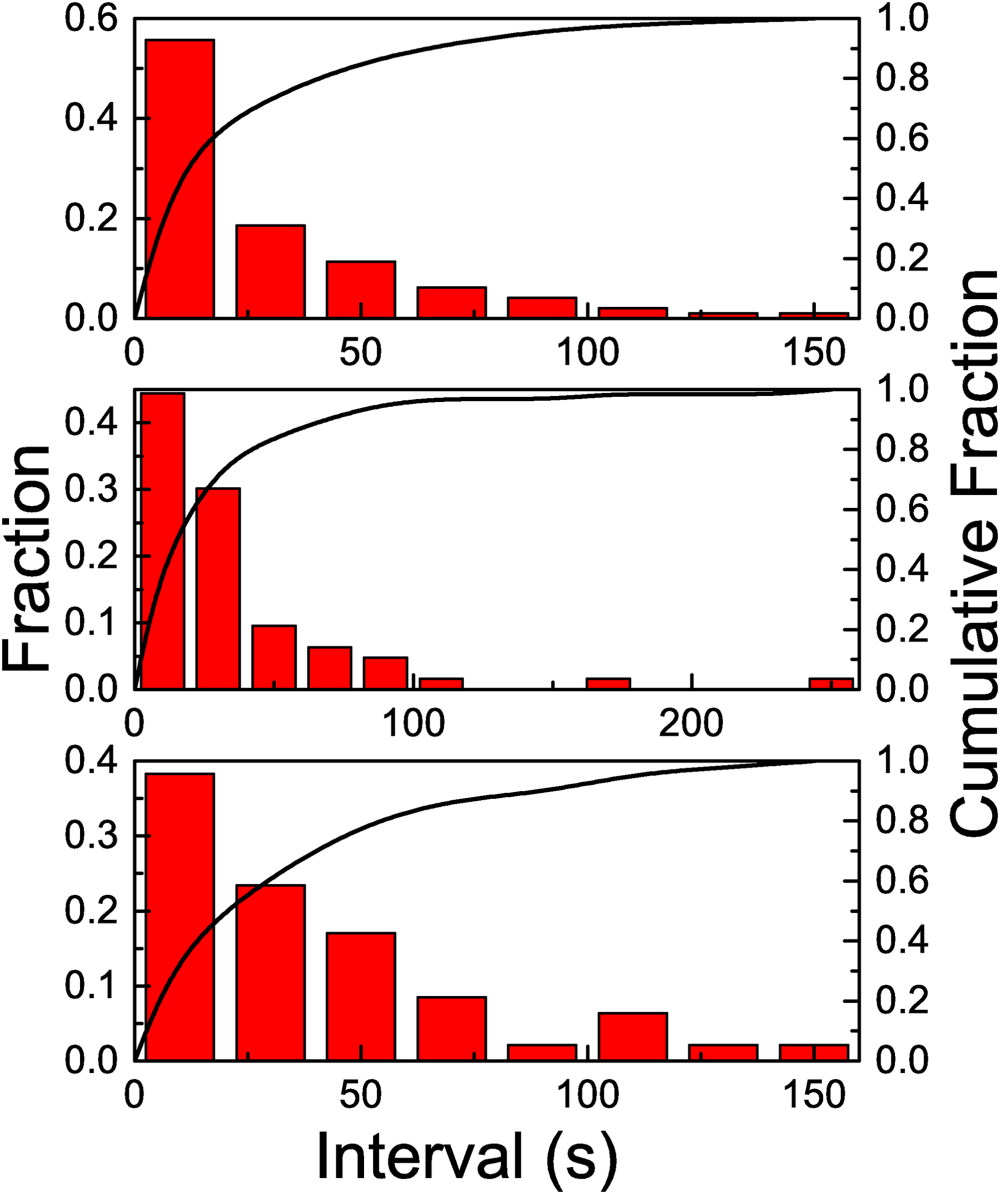
The *x* axis corresponds to the duration of lapses, in seconds. The histograms shows the distribution of the lapses (left *y* axis), while the full line shows the cumulative fraction (right *y* axis). No treatment: when all wasps were allowed to forage on the resource.

To count with a more precise evaluation of the distribution of lapses between consecutive arrivals we have studied its distribution. The analysis of this distribution is displayed in Fig 3, where we show its histogram and the cumulative fraction. We observe that the events,.i.e. lapses between consecutive arrivals, are mostly accumulated around low values while their frequency decays for larger intervals. Still, the interesting feature is not the abundance of short lapses, but, as mentioned before, the arrival of three wasps in a lapse no longer than 8 seconds. We chose this value after the analysis of our data. The origin of these multiple events separated by short lapses could be attributed both to a local enhancement effect or a recruitment mechanism. To discriminate between these two effects we present next the results obtained by applying treatments 1 and 2.

#### Treatment 1

**Fig 4:**
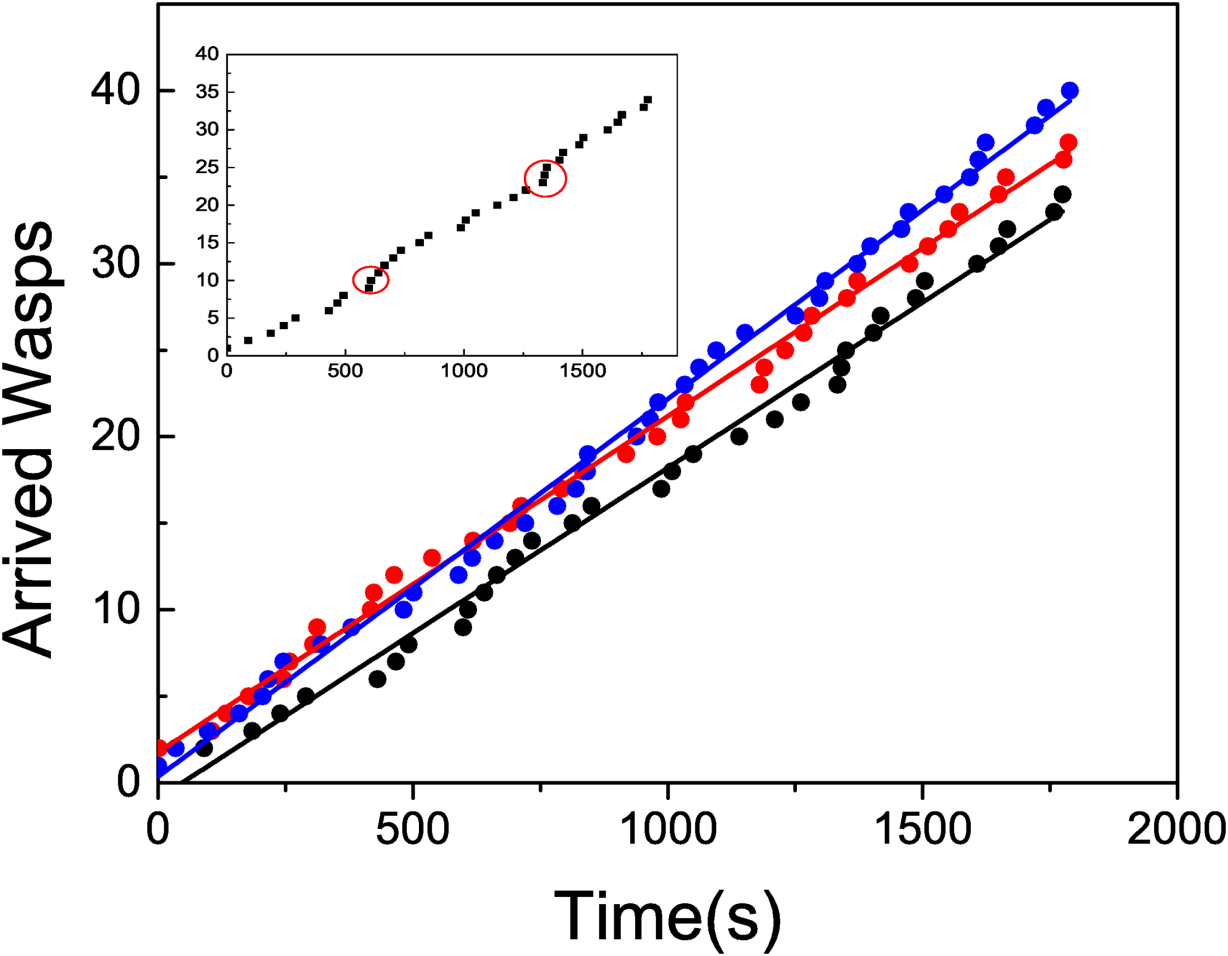
Main plot: Cumulative number of arrived wasps in time, when treatment 1 is applied for several experiments. The full line corresponds to a linear fit. The slopes, measured in unities of wasps/s, are (black) 1.90 10^−2^ ± 3 10^−4^ (*R*^2^ = 0.996) (red) 1.94 10^−2^ ± 2 10^−4^ (*R*^2^ = 0.998) (blue) 2.18 10^−2^ ± 3.4 10^−4^ (*R*^2^ = 0.998). Inset: A particular realization indicating the returns of the marked wasp.

**Fig 5:**
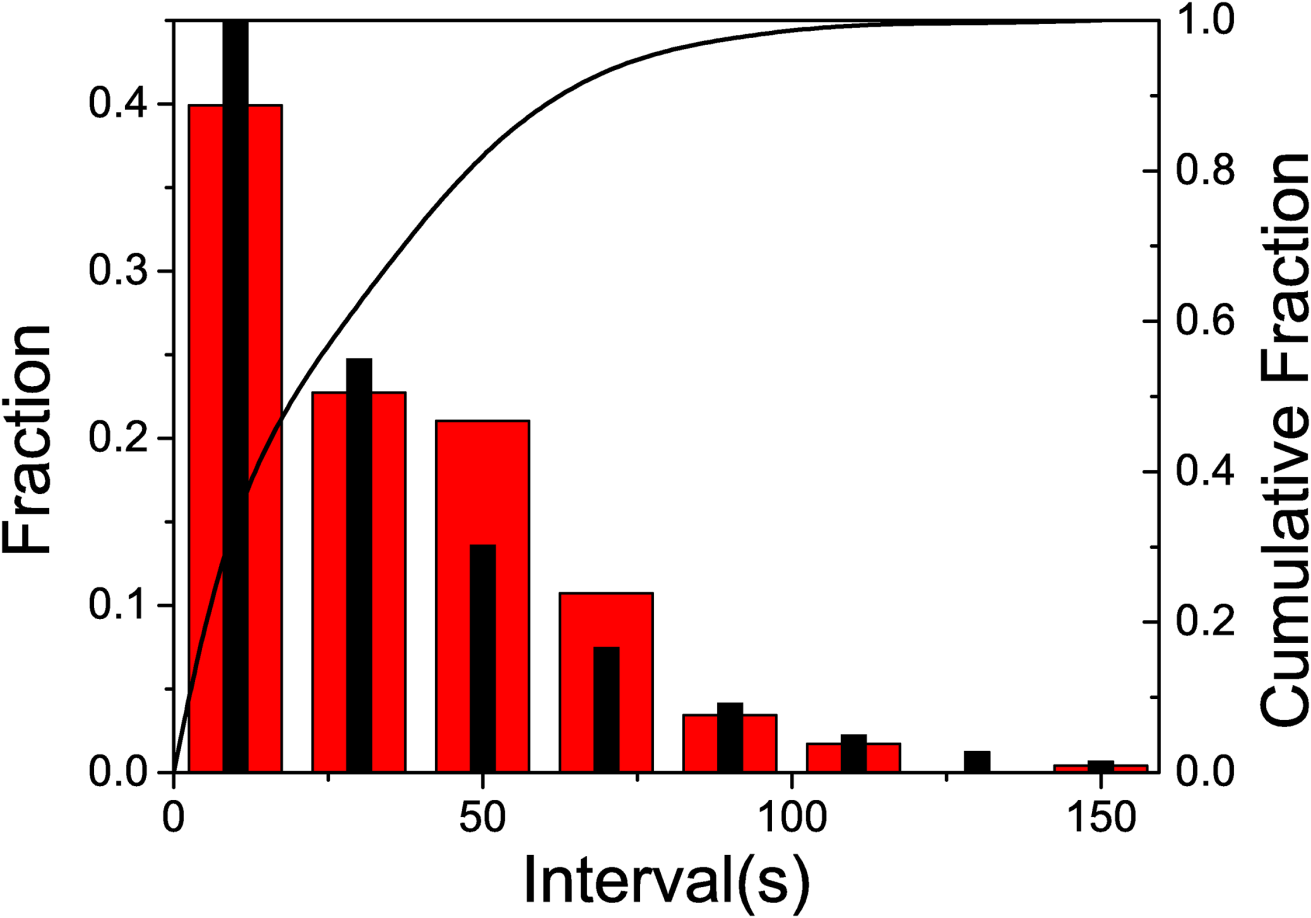
The *x* axis corresponds to the duration of lapses, in seconds. The histogram with wider red columns shows the distribution of the observed lapses, while the histogram with thiner black columns shows the calculated lapses for a Poisson process with *λ* = *λ*_1_. The full line shows the cumulative observed fraction (right *y* axis). Treatment 1: when one wasp was allowed to return to the nest while removing other foragers from the resource.

The situation where one wasp was allowed to return to the nest removing other foragers from the bait (treatment 1), would allow for recruitment while inhibiting local enhancement. Thus, we did not expect an exponential behaviour on the wasps arrival time series, although the presence of simultaneous arrivals would still occur due to recruitment. We performed a similar analysis to the one done for the situation where complete communication was allowed, studying both the time series obtained from recording the arrival of wasps and the distribution of lapses between consecutive arrivals (Figs 4 and 5). When one marked forager was allowed to go back to the nest, we observed a stepwise pattern of wasps’ arrivals in small groups of 3–5 individuals. Though the return of the marked wasp was linked to the occurrence of a train of arrivals, the reciprocal is not true. Fig 4 shows time series corresponding to three experiments, that present a linear behaviour with almost the same slope. The inset shows one particular experiment where the marked wasps returned twice to the feeder. A red circle indicates those moments, and in this case they are in correspondence with train of arriving wasps. As mentioned before, we have also observed some trains not linked to the arrival of the marked wasp.

The calculated mean arrival rate is *λ*_1_ = 2.087 10^−2^ ± 7 10^−5^ arrivals per second. The linear behavior of the curves in Fig 4 imply a constant arrival rate. This linear behavior could be attributed to a homogeneous Poisson process, that would be in contradiction with the hypothesis of recruitment.

A homogeneous Poisson process is associated to the count of events that occur at a constant rate, in our case the arrival of a wasp [49]. It is a stochastic process that is characterized by a rate parameter *λ* such that the number *N* of events in a time interval (*t*, *t* + *τ*) follows a Poisson distribution, with probability

$$\Pi \left[N\left(t+\tau \right)-N\left(t\right)=k\right]=\frac{e^{-\lambda \tau }{\left(\lambda \tau \right)}^{k}}{k!}$$ (1)

where *k* in a natural number. The parameter *λ* is the expected number of arrivals per unit time. The Poisson process assumes that the number of occurrences in non overlapping intervals is independent of each other and that the probability distribution of the number of occurrences in any time interval only depends on the length of the interval. Thus, a recruitment effect that implies a certain degree of correlation between arrivals will produce a measurable deviation from these premises.

In Fig 5 we observe the histogram corresponding to data obtained with treatment 1. Superimposed to this histogram we have plotted another histogram, obtained from a simulated Poisson process (using the same bin size), with *λ* = *λ*_1_, the measured rate. We observe that there is a close agreement between these two histograms, so the abundance of events corresponding to short lapses is not an indication of any correlation.

**Fig 6:**
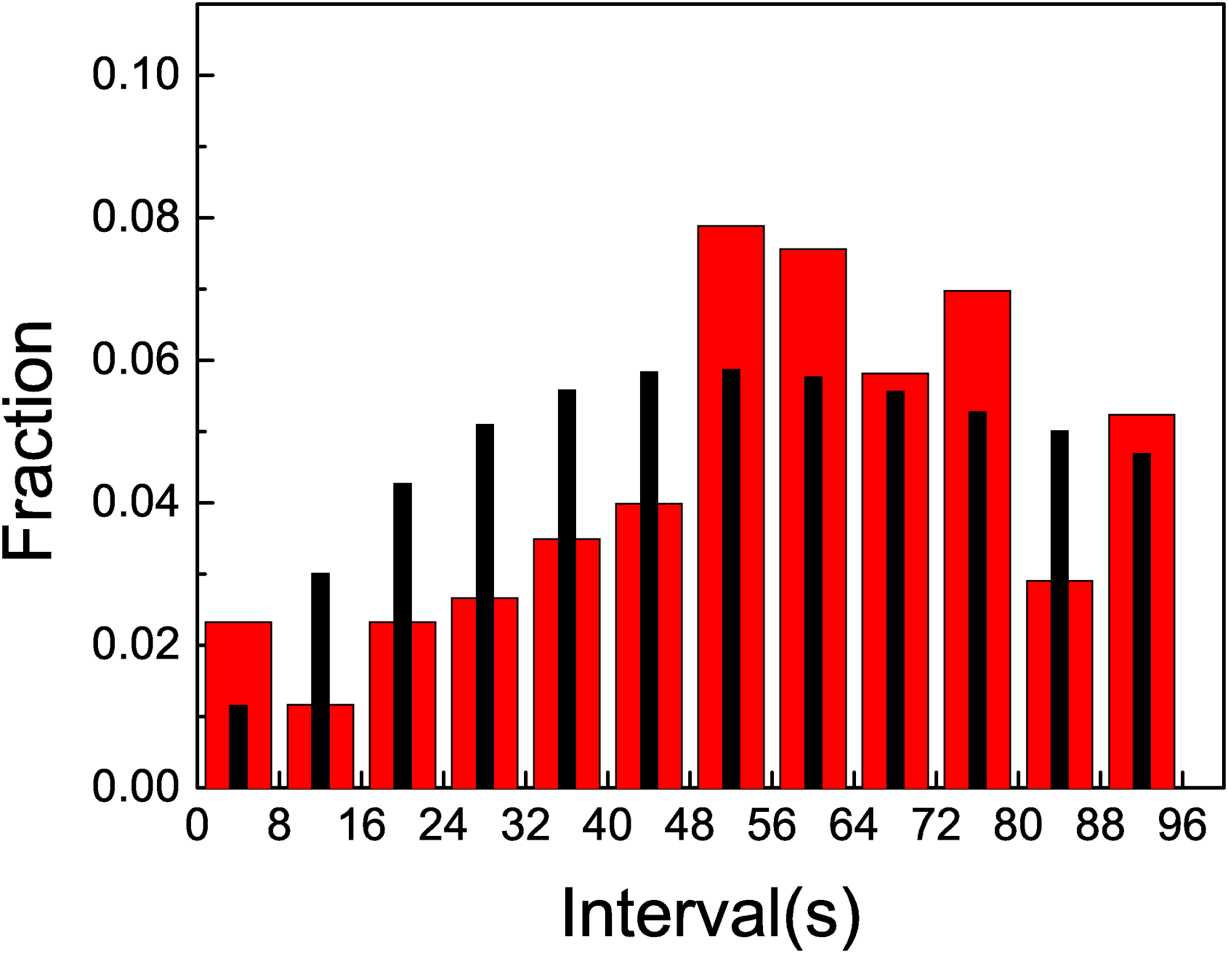
The plot shows a zoom of the histograms corresponding to lapses containing three consecutive arrivals. The histogram with wider red columns shows the distribution of the observed lapses, while the histogram with thiner black columns shows the calculated lapses for a Poisson process with *λ* = *λ*_1_.

The departure from the a stochastic process is evident when we calculate the probability of three consecutive arrivals in a lapse of 8 seconds. The probability of such event when considering a Poisson process is is 1.15 10^−2^ while for the measured data the calculated value is twice higher, 2.2 10^−2^ ± 3 10^−3^. This fact is an indication that this pattern of arrival to the feeder could reflect that recruitment is occurring. In Fig 6 we show a zoom of the histogram of lapses containing three consecutive arrivals. Our interest is centered in the lowest bin, measuring the fraction of three consecutive arrivals within 8 seconds. The experimental data departs from the simulated ones.

The results obtained with treatment 1 show that the emergence of trains of wasps still persist although local enhancement was inhibited.

#### Treatment 2

**Fig 7:**
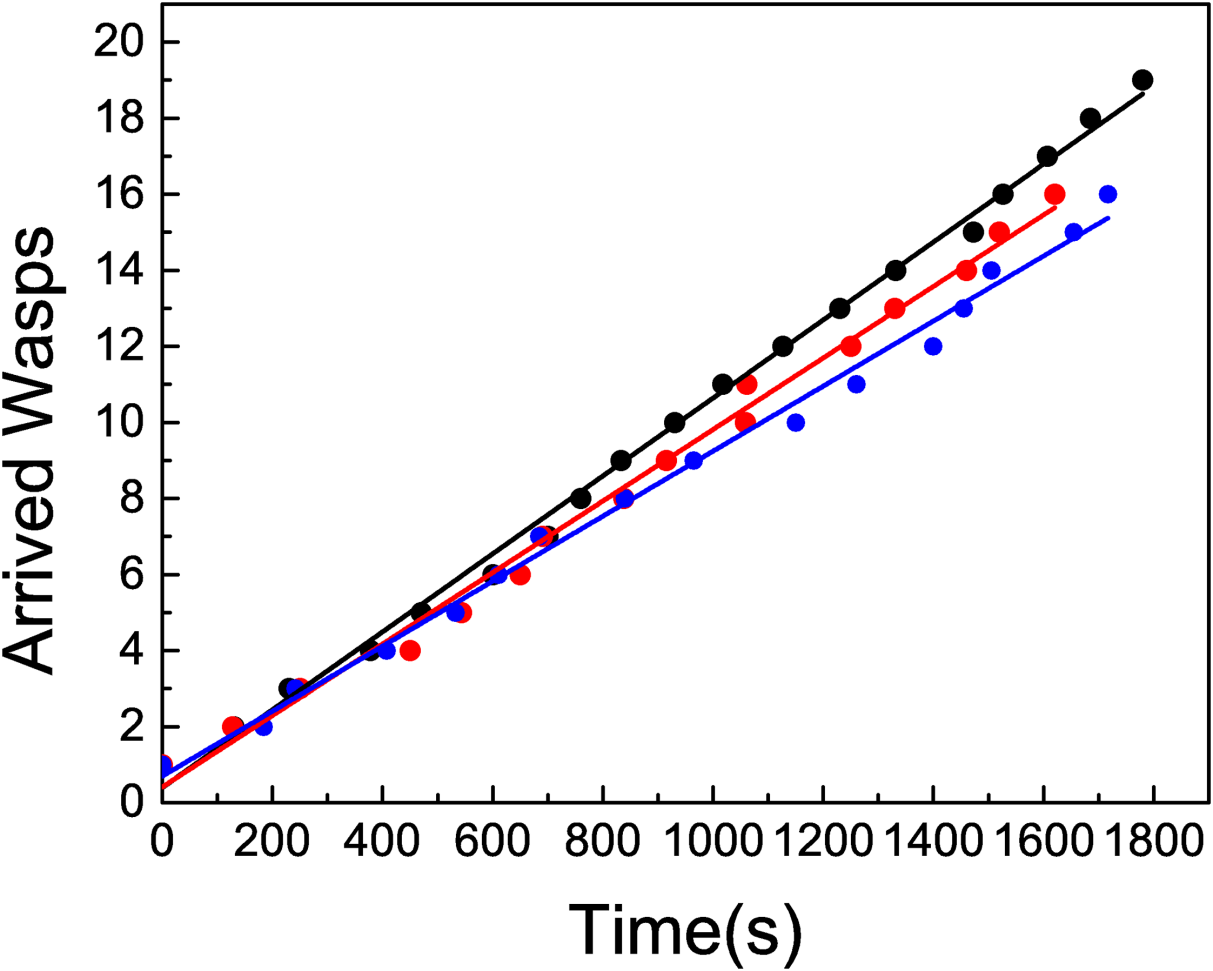
Cumulative number of arrived wasps in time, when treatment 1 is applied for several experiments. The full line corresponds to a linear fit. The slopes, measured in unities of wasps/s, are (black) 1.02 10^−2^ ± 1 10^−4^ (*R*^2^ = 0.998) (red) 9.4 10^−3^ ± 2 10^−4^ (*R*^2^ = 0.997) (blue) 8.5 10^−3^ ± 2 10^−4^ (*R*^2^ = 0.997).

**Fig 8:**
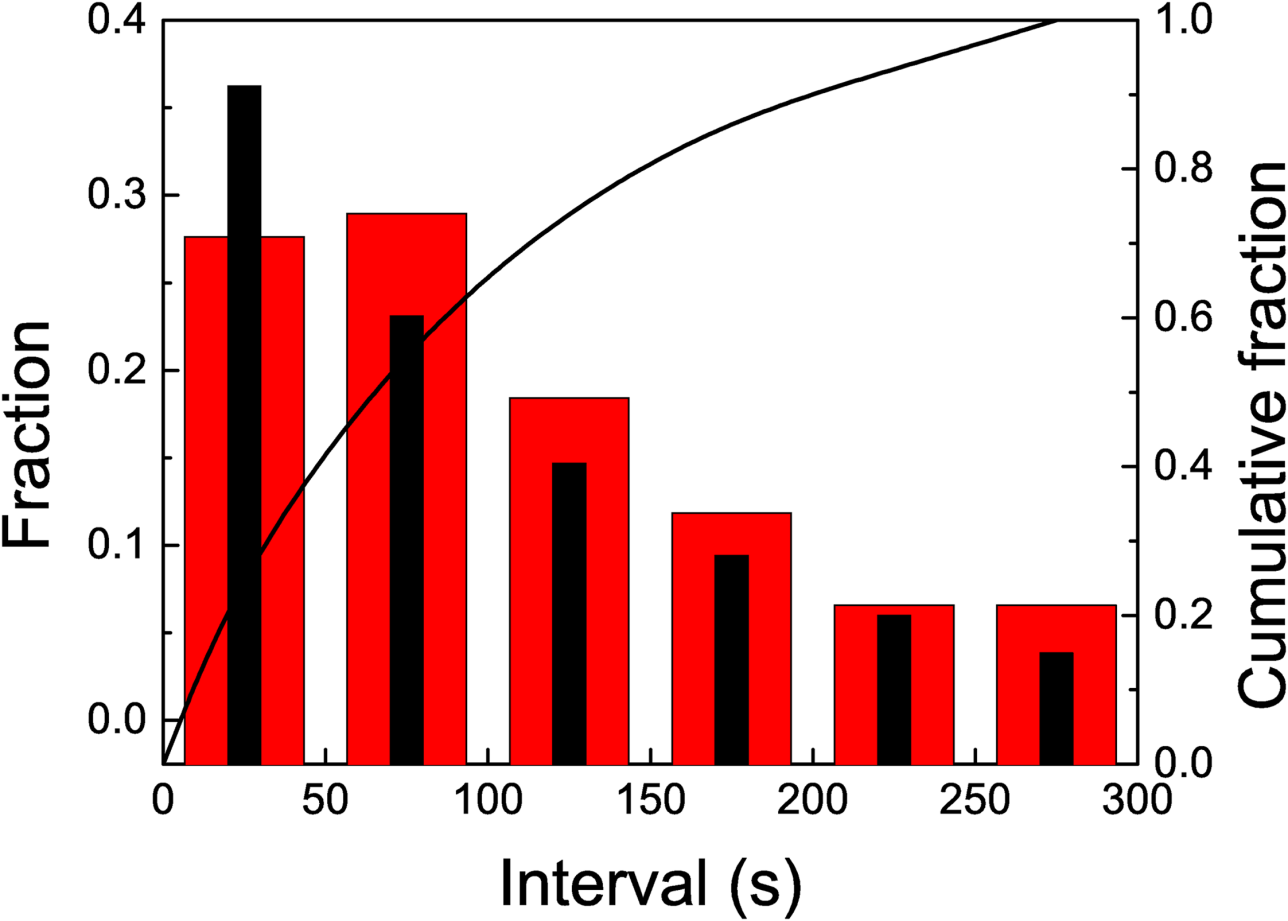
The *x* axis corresponds to the duration of lapses, in seconds. The histogram with wider red columns shows the distribution of the observed lapses, while the histogram with thiner black columns shows the calculated lapses for a Poisson process with *λ* = *λ*_2_. The full line shows the cumulative observed fraction (right *y* axis). Treatment 2: when all foragers were removed as soon as they landed on the resource.

If all foragers are removed as soon as they arrive to the feeder, neither local enhancement nor recruitment would be expected. The results confirm this assumption. In Fig 7 we observe an almost linear arrival pattern. As in treatment 1 we show time series corresponding to three experiments. The time series can be fitted with a linear function by using the least squares methods. From this fit we can extract an arrival rate *λ*_2_ = 9.68 10^−3^ ± 7 10^−5^ arrivals per second, less than the half of the corresponding value for Treatment 1, *λ*_1_ = 2.087 10^−2^ ± 7 10^−5^. The histogram in Fig 8 shows a distribution of lapses qualitatively similar to the previous one (we have considered a larger bin size). The time series does not show any evidence of trains of wasps, as observed in the previous cases. In principle, the arrival pattern of wasps when local enhancement and recruitment are avoided can be linked to a Poisson process. As in the previous case, we have compared the histogram corresponding to our data with the one obtained with a simulated Poisson process with *λ* = *λ*_2_. Again, we observe a close agreement between the two. This time, we have not observed events corresponding to more than two consecutive arrivals in a short lapse, while the probability of the occurrence of such event in the Poisson process is low but not null (2.5 10^−3^). The results suggest that when there is no information transfer occurring at a distance from the resource, while impeding local enhancement, wasps’ arrival rate is continuous and constant. Interestingly, when the information transfer between the nest and the feeder is allowed (i.e., one wasp can return to the nest although all other incoming foragers were removed), the arrival distribution, as well as the total number of wasps arriving to the feeder suggest recruitment. If such a difference is observed by allowing one wasp to travel between the nest and the feeder, the increase could be exponential when all wasps can return, explaining the exponential curve observed when no treatment was applied.

**Fig 9:**
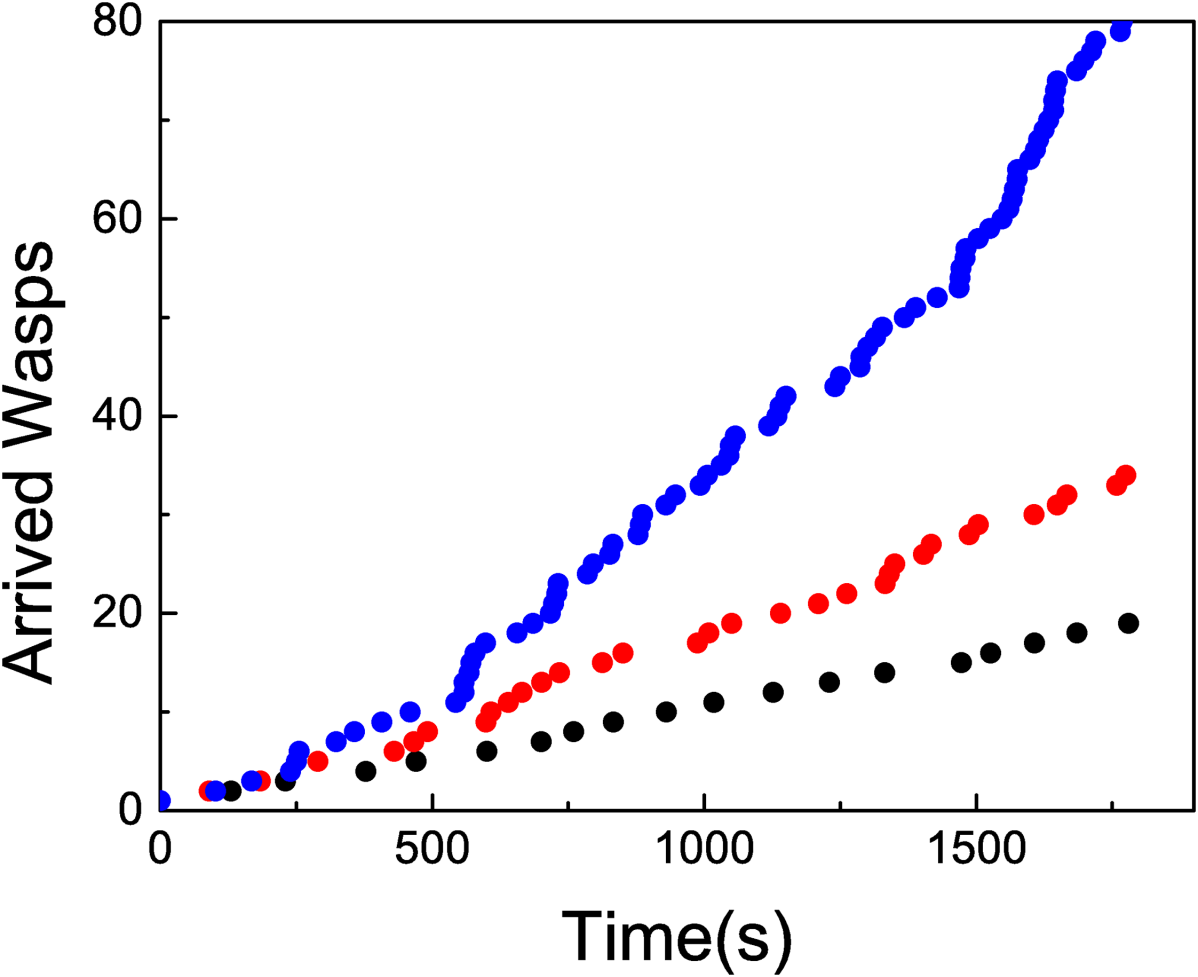
Three typical realizations corresponding to (blue) No treatment (red) Treatment 1 (black) Treatment 2.

In Fig 9 we show a comparison between three realizations associated to each one of the tree protocols. The differences between the three cases are apparent, as well as the non linear behaviour of the control case.

## 5 Discussion

The present study demonstrates the existence of recruitment in *Vespula germanica*, given that a very different pattern of wasps’ arrivals and a higher frequency of wasps’ visits to the resource were observed when communication flow between experienced and naive foragers was allowed. The results showed that recruitment takes place at a distance from the food source in addition to local enhancement. When both local enhancement and recruitment away from the food source were occurring simultaneously, the wasps’ arrival pattern to the feeder was exponential. When only recruitment away from the feeder occurred, the arrival pattern was linear, and the number of wasps arriving was twice as many as when neither communication nor local enhancement were allowed. Moreover, when return to the nest was impeded, wasps’ arrival at the bait was regular and constant.

Typically, when a forager was allowed to leave the feeder with food it was accompanied by one or more wasps on its return to the bait. This behavior agrees with those results reported by Santoro et al [29] for *V. vulgaris* wasps, where occasionally foragers were observed arriving at the bait almost simultaneously (within 3 s) with another individual. The mathematical model applied in our study demonstrated that the probability that a forager returning to the food source was accompanied by other wasps was greater than what would be expected by chance. This was particularly evidenced by the fact that a proportion of the marked wasps arrived together with two other wasps (i.e. three tandem foragers), a pattern not compatible with a Poisson distribution. These tandem arrivals suggest pilot flights, as observed in other flying hymenopterans [28, 50], with newcomers possibly finding their way to the food resources by following experienced nest mates. The present findings suggest that foraging behavior strategies in *V. germanica* and *V. vulgaris* is similar with regard to recruitment. Interestingly, these tandem flights have not been observed or described in other species of vespid wasps [23, 30] and may be associated with the foraging capabilities and invasive characteristics of these two species of wasps. The results obtained demonstrated the occurrence of recruitment away from the resource although we cannot determine whether it was taking place at the nest or during the flight back to the nest. However, previous studies suggest that recruitment tends to take place at the nest [29]. Interestingly, Wilson-Rankin [51] found that twice as many *Vespula pensylvanica* wasps foraged on chicken baits when all foragers were allowed to return to the nest, compared to when visitation was restricted to naïve wasps only. However, in the present study with *V. germanica*, when all foragers were allowed to forage on the bait and return to the nest, the arrival pattern was exponential and the number of wasps was approximately 4 times greater than when no communication was allowed. This discrepancy could be due to the fact that no local enhancement occurs in *V. pensylvanica* [30] in contrast to what has been observed in *V. germanica* [16, 17]. This could be associated with the great invasive properties of *V. germanica*, and points to the significance of local enhancement as a foraging strategy.

Our findings on communication leading to social exploitation of protein-based resources fit in well with the fact that this type of food source (carrion) is ephemeral and its location unpredictable. Nevertheless, similar recruitment patterns have been observed in *V. vulgaris* and *V. germanica* foraging on carbohydrate sources [25, 29], indicating that this behavior is generalized in these two species. This suggests that the foraging strategy which involves social learning by means of recruitment mechanisms and local enhancement would increase their effectiveness at exploiting resources. In conclusion, this is the first study to demonstrate recruitment in *V. germanica* at a distance from the food source by modelling wasps’ arrival to a protein-based resource. The mathematical analysis revealed that the distribution pattern while exploiting a protein based resource is exponential involving tandem arrivals that cannot be interpreted as a random process. These social communication processes together with the complex individual cognitive abilities found in *V. germanica* wasps (e.g. [33, 37, 39, 40]) would enhance their behavioral plasticity contributing to their invasive success.

## Supporting Information

S1 Data No Treatment. (TXT)

S2 Data Treatment 1. (TXT)

S3 Data Treatment 2. (TXT)
